# Early versus delayed reconstruction for bile duct injury a multicenter retrospective analysis of a hepatopancreaticobiliary group

**DOI:** 10.1038/s41598-022-15978-x

**Published:** 2022-07-08

**Authors:** Danny Conde Monroy, Paula Torres Gómez, Carlos Eduardo Rey Chaves, Andrea Recamán, Manuel Pardo, Juan Carlos Sabogal

**Affiliations:** 1 HPB Surgery Department Bogotá, Méderi, Hospital Universitario Mayor, Bogotá, Colombia; 2 School of Medicine, Universidad del Rosario Bogotá, Bogotá, Colombia

**Keywords:** Biliary tract disease, Risk factors

## Abstract

Common bile duct injury is a severe complication. It is related to increased rates of morbidity and mortality. Early recognition and on-time diagnosis plus multidisciplinary management of this disease led by a hepatobiliary surgeon show fewer complications rate and best postoperative outcomes. However, no guidelines exist about the proper time of reconstruction. This study aims to describe the experience of a specialized Hepato-Pancreatic-Biliary (HPB) group and to analyze the outcomes regarding the time of bile duct injury (BDI) repair. A multicenter retrospective review of a prospective database was conducted. All the patients older than 18 years old that underwent common bile duct reconstruction between January 2014 and December 2021 were included. Analysis and description of preoperative characteristics and postoperative outcomes were performed. A reconstruction time-based group differentiation was made and analyzed. 44 patients underwent common bile duct reconstruction between January 2014 and December 2021. 56.82% of the patients were female. The mean age was 53.27 years ± 20.7 years. The most common injury was type E2 (29.55%). Hepaticojejunostomy was performed in 81.81% (of the patients. Delayed reconstruction (> 72 h) was performed in the majority of the cases (75.00%) due to delays in the referral centers or poor condition. No statistically significant difference regarding complications in early or delayed BDI reconstruction. The mortality rate was 2.7% (n = 1). 2-year follow-up bilioenteric stenosis was observed in 7 patients. Biloma showed a statistical relationship with complex bile duct injuries (*p* = 0.02). Bile duct injury is a severe and complex postoperative complication that increases morbidity and mortality rates in the short and long term in patients undergoing cholecystectomy. In our study, there were no statistical differences between the timing of bile duct reconstruction and the postoperative outcomes; we identified the presence of biloma as a statistically related factor associated with complex bile duct injury; however, further prospective or studies with an increased sample size are required to prove our results.

## Introduction

Around 700,000 cholecystectomies are performed annually in the United States^1–4^ and laparoscopic cholecystectomy is currently the gold standard of symptomatic gallstones or acute cholecystitis management^1,2^; Its benefits over open cholecystectomy are fast patient recovery, less postoperative pain, shorter hospital stay, and faster reintegration to daily activities^2,5–7^.

Although surgeons around the world have accumulated experience and the learning curve has become steeper^7^, in the last three decades biliary tree lesions have increased^8^. Iatrogenic biliary duct lesions can occur by either an open or laparoscopic approach and it is associated with significant morbidity and low but not negligible mortality^3,4,9^.

Surgery by laparoscopy shows two to three times higher rates of bile duct lesions (0.4–0.6%) in comparison to an open approach (0.2–0.3%)^5,6,10,11^. Patients who suffer common bile duct injury show increased 1-year mortality of approximately 7.2%, and 14.5% in a 5-year follow-up^12–15^. Additionally, these lesions have an impact on the healthcare system, increasing costs up to 126% after bile duct repair^4,7,10,12,13,16–20^.

To decrease bile duct injuries, risks must be identified both previously and during surgery. A preoperative evaluation of risk factors, early injury identification, and accurate management of bile duct injuries are crucial to decrease morbidity and mortality. Also, it’s well established the importance of the surgeon’s experience and formal Hepatobiliary and pancreatic (HPB) training when treating bile duct injuries. As Halle-Smith et al. described, reconstruction performed by a non-hepatobiliary surgeon increases a 4-time risk to present postoperative complications^14^. Among those relevant factors, time of reconstruction rises in literature as a key component of reduced morbidity and mortality^21^. Early reconstruction is associated with a shorter hospital stay, cost reduction, and reduced burden on the patient. On the contrary, delaying surgical reconstruction allows for optimization of the clinical condition, sepsis control, and delimitation of bile duct ischemia, eventually influencing long-term strictures and bile duct leak^21^. Some reports describe no relevance in the time of reconstruction for the early and delayed complication rates^22,23^. Therefore, this topic is still a matter of debate.

The current study aims to describe the experience in the management of common bile duct injury after laparoscopic/open cholecystectomy by a hepatobiliary group in high complexity centers in Colombia and to describe the association between time of reconstruction and short- and long-term complications.

## Methods

The present study was performed in four centers where the iatrogenic biliary injury occurred (Two academic and 2 non-academic hospitals). A retrospective analysis was done. Our primary outcome is to describe the different outcomes between early and delayed times of the reconstruction. The secondary outcomes were to evaluate the associations between preoperative and intraoperative variables and short/long term outcomes. Biliary reconstruction was performed by the same HPB surgeon. Ethical compliance with the Helsinki Declaration, current legislation on research Res. 008430-1993 and Res. 2378-2008 (Colombia), and the International Committee of Medical Journal Editors (ICMJE) were ensured under the Ethics and Research Institutional Committee (IRB) approval. Institutional Review Board approval (Hospital Universitario Mayor Méderi, Universidad el Rosario) from the institutions involved. Patients over 18 years old who were diagnosed with common bile duct injury following laparoscopic or open cholecystectomy between January 2014 and December 2021, were included.

Demographic characteristics, risk factors, remission time, intraoperative findings, imaging studies performed, type of injury (Strasberg classification system), postoperative evolution, complications based on the Clavien-Dindo classification, and postoperative mortality were included. All patients were followed for postoperative stenosis at 6, 12, and 24 months. Descriptive statistics of all study parameters were provided. Data was analyzed using STATA 17 software^24^. Continuous data was summarized by its mean, median, standard deviation, and interquartile range. Categorical data was summarized by frequency and proportion. Bivariate analysis was performed. Qualitative variables were analyzed using chi-square statistics (Fisher's exact test when appropriate). Quantitative variables were analyzed, based on normality, with Spearman's or Pearson's association’s correlation coefficient accordingly. A multivariate analysis was performed to evaluate the association between clinical variables (Cholangitis, jaundice, and biloma), and the severity of the BDI.

Association among clinical variables and the severity of bile duct injury were evaluated. Complex injuries were defined as Strasberg types E3-E5. Clinical findings such as abdominal pain, cholangitis, biloma, and comorbidities (arterial hypertension and diabetes mellitus type 2) were evaluated. The significant statistical association was defined if a *p*-value < to 0.05 was reached.

Timing of the reconstruction was defined in three groups: intraoperative, early (< 72 h since the BDI) or late (> 72 h). Associations between the timing of the reconstruction and postoperative outcomes such as mortality, bilioenteric anastomotic stricture, ICU requirement, and Clavien-Dindo classification were evaluated. The significant statistical association was defined if a *p*-value < to 0.05 was reached.

### Initial approach

The initial management varies according to the identification of the lesion. intraoperative lesions and availability of HPB surgery are treated in the same procedure by open surgery. If no HPB availability, place of drains and no conversion to laparotomy is advised to the primary surgeon and referral to the HPB group as soon as possible. In the early and late cases, a complete evaluation of the clinical condition of the patient is made before the decision of surgical reconstruction. Depending on the type of injury, endoscopic management is considered in some low complex lesions (Strasberg A, B, C, D) lesions. High complex lesions are managed by surgical reconstruction. A flowchart with the management is displayed in Figure S1 (see “Appendix”).

### Surgical approach

Méderi Hospital is a referral center for HBP complex surgeries in Bogota. Iatrogenic biliary injuries were done in three other hospitals. However, the same HPB surgeon was able to perform reconstruction in all four institutions.

A standardized technique was performed. We make a midline supraumbilical laparotomy. Blunt dissection is performed by using low-energy electrocautery throw embryologic planes. The hepatoduodenal ligament is mobilized and adherences are dissected. The next step is to perform a Kocher maneuver. Right after, dissection of the hilar plate to identify the bile duct injury. Biloma is drained, and clots and old bleeding are removed when needed. The abdominal cavity is cleaned. Healthy bile duct tissue is exposed. Standard transmesocolic hepaticojejunostomy (HJ) is performed using a Roux-en-Y technique either 4–0 or 5–0 polydioxanone suture depending on bile duct thickness. Finally, intestinal transit is reconstructed by two layers of side-to-side jejunojejunostomy with 4-0 polydioxanone at 60–90 cm to the HJ. Either Jackson-Pratt or Blake drain was left based on specific criteria and availability (see Appendix Photos 1 and 2).

### Follow-up

By institutional protocol, follow-up was performed at 6, 12, and 24 months to determine late postoperative complications and postoperative bilioenteric anastomosis stricture. Magnetic cholangioresonance was performed to confirm the suspected diagnosis.

### Ethical approval

All procedures performed in studies involving human participants were in accordance with the ethical standards of the institutional and/or national research committee (Hospital Universitario Mayor Méderi – Universidad el Rosario) and with the 1964 Helsinki declaration and its later amendments or comparable ethical standards.

### Informed consent

Informed consent was obtained from all individual participants included in the study, and reposes in clinical history.

## Results

In our center, 44,424 cholecystectomies were performed between 2014 and 2021. 44 patients with bile duct injury were collected between Hospital Universitario Mayor Méderi (32) and Hospital Universitario San Ignacio (9); one patient from Clínica Palermo and two patients from Clínica Colsubsidio. The 6-year estimated Incidence of bile duct injury was 0.07%. Surgical management, follow up and data analysis was evaluated together for final evaluation.

### Demographic-clinical

A total of 44 patients were included in the study. The mean age was 53.27 years ± 20.7 years. Females constituted 56.82% (n = 25) of the cases. Body Mass Index (BMI) average was a range between 18.5 and 24.9 kg/m^2^ (68.18% n = 30). The initial surgical approach in the primary surgery was mainly laparoscopic (45.45% n = 20), and 18.18% (n = 8) required conversion to laparotomy due to the surgeon’s decision. The Tokyo classification in cholecystitis patients was Tokyo I (15.91%, n = 7), Tokyo 2 (50.00%, n = 22), Tokyo 3 (4.55%, n = 2) and no cholecystitis (29.55%, n = 13). The indications of inserting a Blake drain in 56.82% (n = 25) of patients were biliary leak after finishing reconstruction and previously known biloma. Half of patients were operated at teaching hospitals (50.00%, n = 22). Summarized characteristics are displayed in Table 1 (see “Appendix”).

### Diagnosis

In our series, bile duct injury diagnosis was classified into three groups. During surgery (38.64%/n = 17), at the first 72 h of the lesion (6.82%/n = 3) and late diagnosis after 72 h (54.55%/n = 24). In the first group, 73.91% of bile duct injury was found through intraoperative cholangiography. In the early and late group of patients, confirmation of the injury was made in the postoperative time by cholangioresonance in 47.73% of the cases. 20.45% of the patients followed an Endoscopic retrograde cholangiopancreatography (ERCP) to diagnose and treat the injury (see Table 2 “Appendix”). In the ERCP cases (n = 9), the lesions were identified as highly complex, so endoscopic management was insufficient for bile leak treatment. A routine blood test was made for all patients (total bilirubin, direct bilirubin, alkaline fosfatase, white blood cell count, serum creatinine y partial thromboplastin time). Results are described in Table 3 (see “Appendix”).

### Type of injury

The type of injury was classified according to the Strasberg classification. The most frequent lesion was E2 (29.55%, n = 13). 68.19% of patients had complex biliary tree bifurcation injury (E 3-4), (see Graph 1). In addition, 2 patients were documented with arterial injury, in both cases with right hepatic artery ligation. However, incomplete data was found. No portal injury was documented.

There is not enough information in the surgical report about vascular injuries.

### Timing of the reconstruction

Biliary reconstruction was performed in most of the cases (75.00%, n = 33) in a range between 72 and 120 h after the diagnosis. Two particular cases were reconstructed after a month. One of them due to sepsis and the other one due to administrative delays in referring the patient. Eight patients were taken for reconstruction between 24 and 72 h after diagnosis and only three cases had reconstructive surgery in the same surgical time as cholecystectomy after the general surgeon recognized the injury; a comparison between the timing of surgical reconstruction and patient’s characteristics are displayed in Table 5 (see “Appendix”). Delayed reconstruction in our study (> 72 h) shows no statistical relationship with mortality, bile duct stricture, or severe postoperative complications defined as Clavien-Dindo III–IVa (*p* = 0.12; *p* = 0.08; *p* = 0.42; *p* = 0.54 respectively), with no differences between this approach, and surgical reconstruction in the early post-cholecystectomy period (see Table 6, “Appendix”).

### Surgical reconstruction

In our analysis 81.81% (n = 36) of BDI were reconstructed by hepaticojejunostomy, 6.82% (n = 3) underwent choledocoplasty, and 4 cases (9.09%) were managed with primary closure of the bile duct. One case (2.27%) required a double bilioenteric anastomosis. Intraoperative bleeding was on average 244 cc. The average surgical time was 206.83 min. In the first surgery, a drain was left in 56.82% of the patients with suspected bile duct injury, it is important to point out that patients should have bile duct drains with a stent in situ during surgical injury if it is recognized or subhepatic drains should be placed to avoid biloma or peritonitis. This is a key factor when it is mandated to refer patients to an HPB center.

### Postoperative results

The Intensive care unit (ICU) was required in 7 cases after the surgery period due to cholangitis and the mean ICU stay there was 0.72 days ± 1.62. Postoperative complications were analyzed using the Clavien-Dindo classification. Type 1 represents the majority of our patients with 72.93% (n = 32), followed by type 3B occurring in 6 patients (13.64%), and type 2 with 9.09% (n = 4) of the patients who need a blood transfusion. Types 3A–4A each occurred in 1 patient (2.27%), and only 1 patient (2.27%) required surgical re-intervention to address its complication. No patients showed complications of type 4B. Mortality was present in one case (2.27%), this particular case was reconstructed one month after bile duct injury due to a delay in biliary injury recognition and delay in referral to our HPB center. Bilioenteric stricture was observed in 7 patients after a 2-year follow-up and all of these cases required new bilioenteric reconstruction.

Biloma has a statistical relationship with complex common bile duct injury (*p* = 0.02, CI 95%), the presence of biloma shows a 41-fold risk of presenting with a Type E3-E4 Strasberg injury of the bile duct (see Table 4, “Appendix”).

## Discussion

Since the first description of laparoscopic cholecystectomy in 1985, it has become the gold standard in care for patients with gallstones and acute cholecystitis. Despite the decrease in complications due to a steeper learning curve, the incidence of bile duct injury continues to be one of the most feared complications, currently occurring at 0.2–0.7% of patients undergoing surgery^5^. The early diagnosis and optimal treatment are the cornerstones to prevent bad outcomes or major complications^5^.

A controversial debate on the optimal timing of HJ for BDI still persists in the literature. A wide variety of cut-off times for “early” and “delayed” surgery has been described and no guidelines at present show the proper time for BDI repair^22^. However, early reconstruction is always recommended in HPB centers around the world. In this study, we used the most common classification as early (first 72 h of reconstruction after the BDI diagnosis) and delayed (after 72 h)^23^. No associations in complication rates were found between those cut-off values, and the complication rate was similar in those groups (Intraoperative Group *p* = 0.1, Early group *p* = 0.6, delayed group *p* = 0.5). Some recent reports support this finding, a collaborative retrospective study from the European-African HPB Association concluded that the timing of reconstruction did not have an impact on severe postoperative complications, reinterventions, or mortality^25^. As well, Stewart and Way found in a multivariate analysis that the time of reconstruction was not an important factor^26^. However, Schreuder et al. show that reconstruction between 2 and 6 weeks has an increased rate of postoperative morbidity and hepaticojejunostomy stricture^21^. Comparisons between patients’ characteristics and timing of the reconstruction were performed, and no significant differences were found in groups, only bilirubin levels were higher in patients that underwent surgery > 72 h, but this difference is expected in this pathology, based on our data, the timing of reconstruction is not related to increased postoperative complications, mortality, or anastomotic stricture, and suggests that “early does not mean better” in BDI reconstruction, however, a delay of more than 4 weeks could be detrimental in postoperative outcomes^21,25^.

Several risk factors must be revised in the preoperative time to classify the risk of potential complications. There are well-described factors in the literature such as acute cholecystitis and cholangitis that are associated with more complex injuries (Type E) with statistically significant values^27^. In this group, there was a higher rate of biliary duct lesions in patients with acute cholecystitis; 70.45% of the cases had acute cholecystitis at the time of the injury. However, it was not clearly associated with the complexity of the CBD injury (*p* = 0.06). Reasons given for this association may be related to the degree of inflammation, the development of fibrosis, and adhesion processes, which hinder the adequate exposure to the surgical field and cause a modification in the anatomy that prevents an adequate identification of the structures that need surgical repair^28^.

When occurred, identification of the bile duct injury is still an enormous concern, only one-quarter of injuries are recognized during surgery^29^. In this report, 38.64% of BDI were identified in the intraoperative time. Most of the lesions were identified during the postoperative time, the presence of symptoms such as fever, postoperative jaundice, and unmanageable abdominal pain should alert the possibility of bile duct injury^7^. In this study, the most common symptom was unmanageable postoperative pain in 75.00% of the patients. Jaundice appeared in 65.91% of cases and 20.45% of patients presented cholangitis^7^.

The experience of a hepatobiliary surgeon and a specialized group has proven to be relevant to ensure optimal results. 75% of surgeons attempt to repair the injury on their own with a poor success rate of 17%^5^. The impact of bile duct injuries is high, this generates an increase in hospital stay and care costs. Also, the emotional toll of this complication is high, not only for the patient and their family but also for all the nursing staff and the surgical team^26^. Based on a significant number of publications, the international organizations for HPB surgeons (AHPBA, IHPBA) state that bile duct reconstruction must be performed only by HPB surgeons.

Long-term follow-up data for this type of injuries is scarce given the low frequency of presentation and the low publication of case series in our setting^17^. In a series of 400,000 cholecystectomies, Sinha et al. found that biliary tree injuries had a sixfold increased risk of mortality at 1 year compared to those without bile duct lesions^20^. One of the most frequent complications in long-term follow-up is anastomotic stenosis. In our series, 7 patients presented biliary stricture in long-term follow-up established at two years.

The presence of biloma in early or late BDI detection showed statistical association with severity of the injury (p 0.02), with a 41.7 increased risk-fold to present highly complex common bile duct injuries defined in this study as type E3-5 Strasberg lesions.

Vascular compromise is a well-described characteristic associated with complications, however in our patients, there were only 2 cases with confirmed vascular compromise, this finding can be associated with an underreported description in the involved hospitals.

BDI reconstructions are demanding procedures, for this the implementation of minimally invasive surgery (MIS) has been cautiously proceeding. Some of the causative factors are the severely altered anatomy, intra-abdominal collections, adhesions and the previous manipulation and clipping in the initial surgery. Some specialized centers have reported promising results with the reconstruction by minimally invasive surgery. A recent systematic review in Italy described outcomes of MIS of 218 patients in 31 studies. describing postoperative morbidity of 24%, 6% reoperation, and 12% of major complications. nevertheless, with a clear absence of high-quality studies and a high risk of selection bias. Therefore, the impact of minimally invasive surgery is still a matter of research^30^. The role of percutaneous management is also anecdotical; usually its purpose is decompression and derivation of the bile duct^31–34^. For cases with bile duct strictures at a high level, bile duct dilatation could be performed; the success rate of these procedures ranges between 27 and 89%. Morbidity varies between 2.5 and 15% and could include sepsis, bleeding, subcapsular hematoma, or death^30,35–38^. However, this approach depends on the severity of the injury and clinical condition of the patient and should be performed in selected cases in order to avoid morbidity and mortality^30,39,40^. The use of those techniques is limited in our center.

Strengths identified in our study are management by the same hepatobiliary group in multiple institutions and the possibility of follow-up at 30 days and 24 months postoperatively. Results in this study should be interpreted in light of several limitations. Only surgical nature of patients undergoing reconstruction as we did not have data on the total incidence of bile duct lesions (lesions with successful endoscopic approach or patients who died before reoperation), as well as the retrospective nature of the study and the small number of patients included. Finally, a possible underreported description of associated complications like the vascular compromise could influence the results.

## Conclusion

Bile duct injury is a severe and complex postoperative complication that increases morbidity and mortality rates in the short and long term in patients undergoing cholecystectomy. In our study, there were no statistical differences between the timing of bile duct reconstruction and the postoperative outcomes; we identified the presence of biloma as a statistically related factor associated with complex bile duct injury; however, further prospective or studies with an increased sample size are required to prove our results.

## Supplementary Information

Supplementary Information.

## Data Availability

The datasets used and/or analysed during the current study available from the corresponding author on reasonable request due to data protection protocol of the institution.
