# Supplementary material for: Early versus delayed reconstruction for bile duct injury a multicenter retrospective analysis of a hepatopancreaticobiliary group
Source: Sci Rep. 2022 Jul 8;12:11609. doi: 10.1038/s41598-022-15978-x (PMC9270444; doi:10.1038/s41598-022-15978-x)

**Table 1.** Demographic and Clinical Characteristics

| **Variable** | **Value** |
| --- | --- |
| Age | 53.27 (18-87) |
| Female (n) | 56,82% (25) |
| Diabetes mellitus (n) | 15,91% (7) |
| Arterial Hypertension (n) | 36,36% (16) |
| Jaundice (n) | 65,91% (29) |
| Cholangitis (n) | 20,45% (9) |
| Abdominal Pain (n) | 75,00% (33) |
| Biloma (n) | 29,55 (13) |

Quantitative variables displayed as median (Range)

**Table 2**. Imagenologic diagnosis

| **Variable** | **Value % (n)** |
| --- | --- |
| Ultrasonography | 2,27% (1) |
| CholangioResonance | 47,73% (21) |
| Contrasted Magnetic Resonance | 11,36 (5) |
| Computed Tomography | 2,27% (1) |
| Endoscopic Retrograde Cholangio Pancreatography | 20,45% (9) |
| Non image performed | 15,91% (7) |

**Table 3.** Laboratory test

| **Variable** | **Value** |
| --- | --- |
| Total bilirubin median(range) | 4.58 ng/dl (0.39-20) |
| Direct bilirubin | 3.39 ng/dl (0.19-15) |
| Alkaline Phosphatase | 406.48 ng/dl (62-2225) |
| White Cell count | 11.89 /L (3.8-37.3) |
| Creatinine | 0.77 mg/dL(0.3-2) |
| Coagulation test | 1.55 (0.9-5) |

Quantitative variables displayed as median (Range)

**Table 4.** Statistical Analysis

| **Variable** | **Relative Risk** | **P value (CI 95%)** |
| --- | --- | --- |
| Cholecystitis | 0.15 | 0.06 |
| Arterial Hypertension | 8.8 | 0.1 |
| Diabetes Mellitus | 0.2 | 0.3 |
| Jaundice | 0.7 | 0.8 |
| Cholangitis | 0.3 | 0.4 |
| Abdominal pain | 3.73 | 0.3 |
| Biloma | **41.7** | **0.02** |
|  |  |  |

**Table 5.** Comparison between the timing of reconstruction and patients’ characteristics

| **Variable n (Total)** | **Intraoperative reconstruction**  **(Total = 3)** | **24-72 hours**  **(Total = 8)** | **> 72 hours**  **(Total = 33)** |
| --- | --- | --- | --- |
| Female | 2 | 1 | 8 |
| Jaundice | 1 | 5 | 23 |
| Cholangitis | 0 | 1 | 8 |
| Abdominal pain | 0 | 5 | 28 |
| Biloma | 0 | 1 | 12 |
| ICU Requirement | 2 | 0 | 5 |
| Cholecystitis | 2 | 4 | 25 |
| No - Cholecystitis | 1 | 4 | 8 |
| Inotropic requirement | 1 | 0 | 2 |
| Blood transfusion requirement | 1 | 1 | 1 |
| **Variable mean (SD)** |  |  |  |
| Creatinine | 0.66 (0.14) | 0.9 (0.12) | 0.77 (0.06) |
| White blood cell count | 13.3 (2.21) | 11.41 (2.03) | 12.23 (1.07) |
| Total Bilirubin | 1.26 (0.39) | 2.5 (0.75) | 4.9 (0.86) |
| Coagulation test | 1.26 (0.03) | 1.3 (0.12) | 1.5 (0.14) |

**Table 6**. Statistical analysis between timing of reconstruction and outcomes.

| Timing of reconstruction | Intensive care unit stay    n (p value) | Mortality | Clavien Dindo III | Clavien Dindo Iva | Bilioenteric anastomotic stricture |
| --- | --- | --- | --- | --- | --- |
| group 1 Intraoperative  (n = 3) | 2 (0.7) | 0 (0.2) | 1 (0.12) | 0 (0.12) | 1 (0.54) |
| group 2  24-72 hours  (n = 8) | 0 (0.5) | 0 (0.1) | 1 (0.45) | 0 (0.60) | 2 ( 0.15) |
| group 3  > 72 hours  (n = 33) | 5 (0.8) | 1 (0.1) | 5 (0.44) | 1 (0.45) | 4 (0.07) |

_
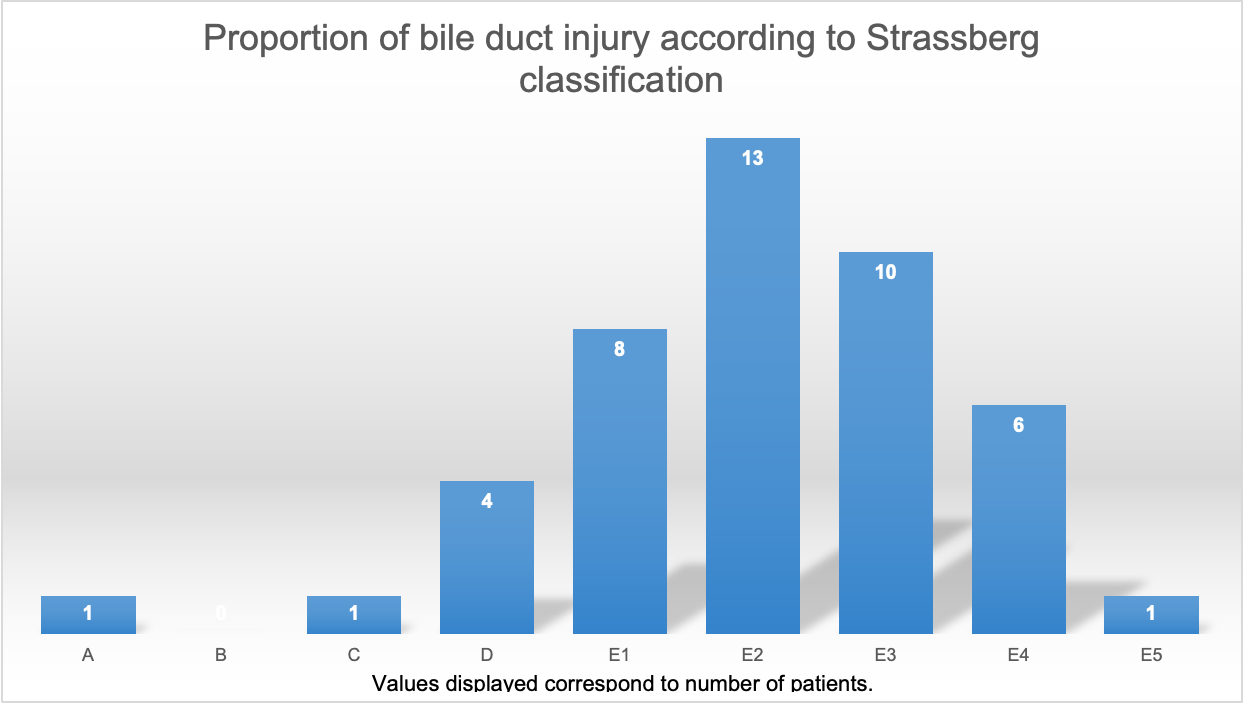
_

Graph 1. Proportion of bile duct injury according to Strasberg Classification .


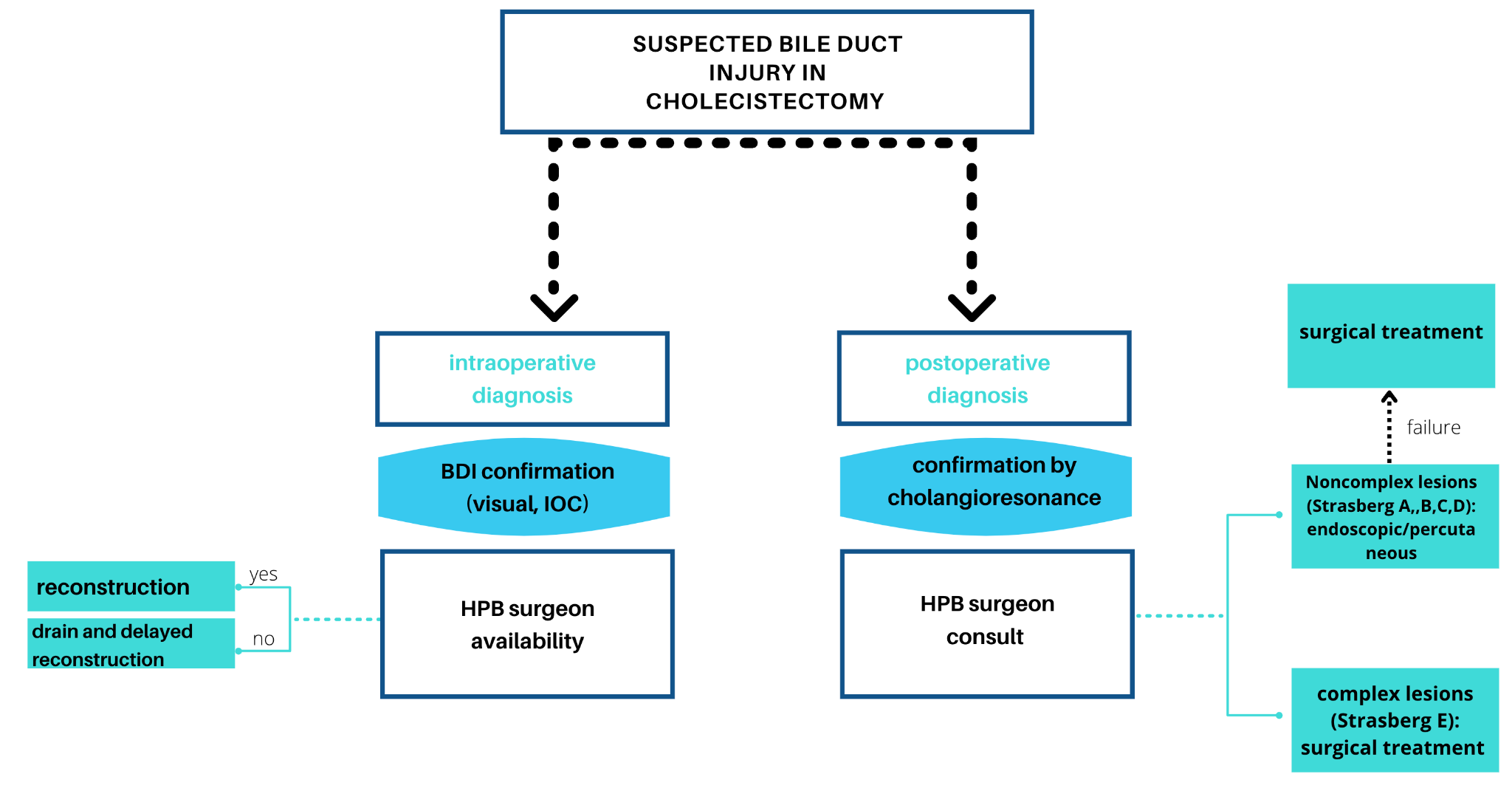


Figure 1. Flow chart of BDI management


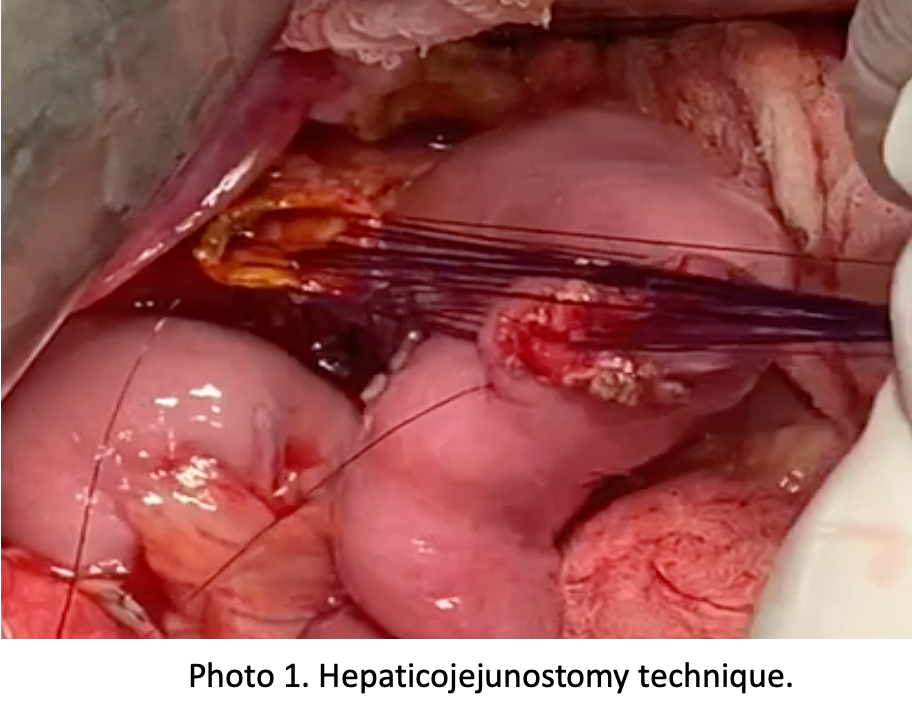


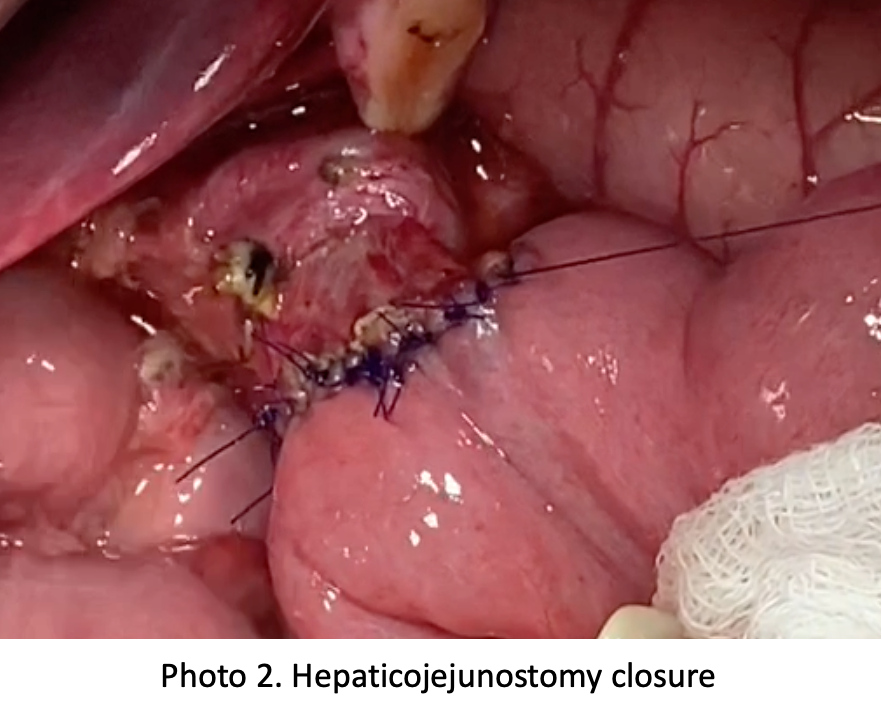

Supplement: Supplementary file 1 — Supplementary Information. [file 41598_2022_15978_MOESM1_ESM.docx]
